# The Influence of Message Framing on Residents’ Waste Separation Willingness—The Mediating Role of Moral Identity

**DOI:** 10.3390/ijerph19105812

**Published:** 2022-05-10

**Authors:** Wei Li, Si Chen, Zhihao Wang, Guomin Li, Xiaoguang Liu

**Affiliations:** School of Economics and Management, Taiyuan University of Technology, Taiyuan 030024, China; chensi0804@link.tyut.edu.cn (S.C.); wzh9204@163.com (Z.W.); liguomin@tyut.edu.cn (G.L.)

**Keywords:** message framing, moral identity, waste separation willingness

## Abstract

With serious environmental problems increasing, waste separation has drawn much attention. Message framing is an important way to popularize separation knowledge and increase people’s separation willingness. Message framing was classified into positive and negative frames in this study, and then based on moral identity theory from the social cognitive perspective, two dimensions of moral identity were introduced as mediating variables to construct a mechanism model of the influence of message framing on waste separation willingness. After a comparative study of three groups of subjects (N = 604), the following conclusions were drawn: (1) message framing positively influenced moral identity and waste separation willingness; (2) both positive and negative message framing positively influenced waste separation willingness through the partial mediating role of moral identity symbolization and internalization; and (3) the mediating role of symbolization was stronger in the effect of positive message framing on waste separation willingness, while the mediating role of internalization was stronger in the effect of negative message framing on waste separation willingness. The findings provide significant information for organizations to effectively carry out message strategies.

## 1. Introduction

With the rapid development of the economy, the amount and complexity of municipal solid waste (MSW) has also increased rapidly [1,2]. Due to the failure to dispose of waste in a timely and effective manner, environmental pollution has become increasingly serious, and people’s health has been threatened [3]. Waste reduction and recycling can be achieved by separation at the source, which greatly reduces the transportation cost and the difficulty of terminal disposal of waste [4,5,6]. As the main actors in source waste separation, residents’ willingness is an important factor related to the performance of waste management [7].

A lack of recycling knowledge is an important cause of the low efficiency of household solid waste recycling, and the willingness to separate and recycle can be effectively promoted through enhanced information publicity [8,9]. Therefore, many countries and regions have vigorously launched information publicity activities to popularize separation knowledge among residents to improve their environmental awareness and separation willingness [10]. For example, Japan strengthened its public message strategy on environmental protection for the public, which led to waste being separated and reduced at the source [11]; Germany [12] and Italy [13] strengthened their policy advocacy to help residents understand the reward and punishment system to enhance their awareness of waste separation. Hence, a message strategy is a prerequisite of and an important influencing factor for residents to acquire knowledge of separating and raise environmental awareness [14,15,16]. However, some studies have come to the opposite conclusion, suggesting that not all message strategies can achieve the expected results. For example, Chen et al. [17] found a negative effect of waste separation publicity on the awareness rate through an interaction effect test. As the frequency of the message strategy increases, residents receive less differentiated information, resulting in “aesthetic fatigue”, which is not conducive to improving separation awareness. To theoretically explain this contradictory phenomenon, the mechanism of the influence of the message strategy on the willingness to separate waste needs to be adequately explored.

Morality is generally accepted by society as a code of conduct and norms and is a good habit of choice that is prosocial and altruistic [18]. Therefore, as a kind of pro-environmental behavior, waste separation is related to morality and can be regulated by moral norms [19,20,21]. A message strategy focused on a subject can change individuals’ moral perceptions associated with this subject [22] and further influence their beliefs, thoughts, and behaviors [23]. Moral identity is the basis of an individual’s self-concept formed by the combination of moral principles, moral concerns, and moral goals [24]. The stronger an individual’s moral identity is, the stronger his or her willingness to behave morally will be [25]. Through a questionnaire survey of three generations of Americans, Rozin and Singh [26] found that compared to those who perceived smoking as a health hazard, people who perceived smoking as immoral were more disgusted with smoking and less willing to smoke. Smith et al. [27] found that college students who viewed cheating as unethical were more likely to prepare for exams rather than cheat on exams. As a means of external stimulus (preintervention), a message strategy can increase people’s understanding of a certain issue through the information channels of public communication and form or change their moral identities. If a kind of behavior that is originally morally neutral gradually becomes morally distinctive after a continuous message strategy, people will form a corresponding moral identity, and a behavioral willingness will increase or decrease the behavior [28]. Pakpour et al. [29] found that a public message strategy improved the moral cognition of Iranian citizens, which in turn increased their willingness to separate. Chan and Bishop [30] claimed that in Western Australia, publicity activities increased young people’s willingness to recycle waste through meeting their moral requirements and enhancing their moral responsibilities. Consequently, moral identity is an inherent mechanism by which a message strategy can influence the willingness to separate waste.

However, this mechanism has a certain complexity. First, message framing is diverse, and it can be divided into positive, neutral, and negative frames from the content. Positive message framing advocates positive aspects, which can stimulate audiences’ positive emotions and thus inspire them to behave in a specified way. For example, residents of city C in Shanxi Province actively participated in separation activities, which made the participation rates of local pilot MSW higher than 80% and achieved greater environmental benefits [31]. Neutral message framing publicizes separation knowledge, policies, and issues in an objective manner to provide citizens with scientific knowledge and real-time news. Negative message framing reveals immoral aspects, which stimulate negative emotions such as disgust and psychological states such as sympathy and moral thinking. It plays a warning role in reducing audiences’ willingness to behave badly [32]. For example, children in low- and middle-income countries were the ultimate victims of the vicious circle in which the economy suffered again from the huge costs caused by pollution and diseases. Such information warns people of the urgency of protecting the environment [33]. The emotions aroused by message framings are various but all impact upon behavioral willingness. Moreover, from the perspective of social cognition, moral identity is divided into two dimensions: symbolization and internalization [34]. The private internal dimension shows the importance of moral traits in the self-concept, and the public symbolic dimension reflects the desire to show moral traits to others through actions [35]. Although both internalization and symbolization positively affect pro-environmental behaviors, they have different impacts [34].

**Figure 1:**
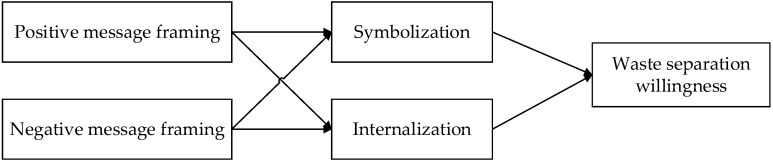
Research framework.

In summary, mediated by moral identity, the influence of different message framing on the willingness to separate waste is complex. However, it is certain that when residents realize that waste separation is about personal morality, they want to consistently separate waste [36]. In this paper, to explore and compare the various mechanisms, two dimensions of moral identity are used as mediating variables to construct a model of the influence of message framing on residents’ waste separation willingness (Figure 1).

## 2. Hypothesis

Neutral message framing is about objective popularization of content such as scientific knowledge and policies and is used to provide audiences with real-time information and a knowledge basis for a certain behavior [37]. As behavior guidance, it can be combined with positive and negative message framing to increase behavioral intention [36]. In addition, positive and negative messages convey distinctly different contents and emotions, so we explore the role of only positive and negative message framing.

Many studies on moral identity are mainly divided into two perspectives: the personality perspective, and the social cognitive perspective. Moral identity from the personality perspective is stable and persistent and mainly reflects the degree of difference in which individuals regard morality as the center of self-consciousness. Thus, it is primarily used to explain moral paradigms or behaviors that are performed after in-depth thought [38]. In contrast, moral identity from the social cognitive perspective is an ordered cognitive schema about moral values, behaviors, characteristics, and goals that is more capable of explaining daily moral behaviors without much thought [35]. Since waste separating is a spontaneous daily “trivial matter” without computation and reasoning, moral identity in this paper is discussed from the social cognitive perspective.

Moral identity is one of the important parts of the self-concept [39], which guides individuals to think and act in a manner consistent with their own identity through the consistency principle [40,41]. When thoughts or actions violate moral codes, people feel a strong sense of incongruity or guilt. To achieve harmony with identity, people need to “correct” the way they act [42]. Moreover, moral identity varies among individuals and changes with situational factors. People with stronger senses of morality are more likely to demand themselves to act in accordance with moral norms, and their willingness to act morally is stronger and more sustained [25,43]. According to self-importance, Aquino and Reed [34] divide moral identity into two dimensions: internalization and symbolization. Internalization corresponds to the inner self, which shows whether an individual violates his or her conscience when doing something. Symbolization is based on the public self, which reflects the tendency of individuals to display their own moral characters through a certain behavior. Winterich et al. [44] found that internalization provided a strong motivation for prosocial behavior, but symbolization usually did not provide additional behavioral motive. However, for people with low internalization, when they perceived that a behavior will be recognized by the public, symbolization will become the primary motive for acting, whereas Wan [45] considered that symbolization could make individuals more willing to act morally, and its role was more important than internalization. Although the role of the two dimensions on moral behavior is still inconclusive, it is certain that as one of the self-judgment concepts, moral identity reflects the level of self-identity of moral traits and inevitably impacts upon an individual’s behavior.

Therefore, when message framing makes individuals realize that waste separation is a moral issue, this message framing mechanism is incorporated into the category of moral internalization and symbolization, and then it positively affects separation willingness through moral identity.

### 2.1. Effect of Positive Message Framing on Waste Separation Willingness—The Mediating Role of Moral Identity

Positively framed messages emphasize the benefits gained from participating behaviors, including collective and individual benefits. From an altruistic perspective, environmentally friendly behaviors such as waste separation, energy conservation, and emission reduction directly benefit resource conservation, pollution reduction, and biological and environmental protection [46]. From a personal perspective, this kind of behavior could build a good personal image by displaying moral traits publicly [47,48]. Positively framed messages display praise and significance of waste separation, which makes the audience reach a consensus on waste separation; they can show personal moral qualities. Moreover, moral identity will be activated in this high-level social consensus.

The internal dimension of moral identity reflects the extent to which moral traits are central to the self-concept [34,49]. When people believe that certain qualities or behaviors are consistent with internal moral evaluations of the self, having these qualities or behaviors will make them feel good [49]. Otherwise, they will suffer psychological discomfort, such as disgust, guilt, and anger [40]. On the other hand, symbolization is about self-realization in public society. Whether external evaluation of moral characteristics is consistent with the “external self” they pursue is the criterion by which individuals decide whether to engage in certain activities [50,51]. Individuals are more willing to act when an action can be recognized or praised by others [52,53,54]. Therefore, moral identity has a promoting effect on waste separation that conforms to ethical standards.

Through positive message framing, both audiences’ internalization of waste separation and separation willingness are increased, because altruism of the message is in line with their inner self requirements and behavioral principles [55]. At the same time, separation activities are prosocial behaviors that conform to social norms and values and correspond to the external moral self-image [56,57]. Most of these activities involve multiple people participating, which enables individuals to demonstrate personal moral qualities and construct their self-images through waste separation in interpersonal interactions [58,59]. Therefore, positive message framing also increases separation willingness by symbolization. In summary, two hypotheses are proposed as follows:

**Hypothesis** **1** **(H1).** *Positive message framing improves waste separation willingness by positively affecting internalization*.

**Hypothesis** **2** **(H2).** *Positive message framing improves waste separation willingness by positively affecting symbolization*.

### 2.2. Effect of Negative Message Framing on Waste Separation Willingness—The Mediating Role of Moral Identity

Negative framing emphasizes the adverse consequences of not performing behaviors [60,61]. In the case of negatively framed separation messages, the contents are mostly textual or pictorial messages about environmental destruction and threats to human health, which could trigger negative emotions [22,62,63]. It has been shown that compared with positively framed messages, negatively framed messages are more likely to trigger negative emotions such as fear, sadness, anxiety, and certain physiological responses more directly, distinctively, and infectiously and then cause cognitive and social responses [64,65,66]. Through the measurement of 722 samples, Grob [67] found negative emotions to be influenced by values and to significantly predict environmentally friendly behaviors. Tapia-Fonllem et al. [68] suggested that negative emotions such as anger significantly affect pro-environmental behaviors such as energy conservation and emission reduction, green purchasing, and recycling. When individuals evaluate their own or others’ behaviors according to social norms, emotions related to social and personal interests are generated, and these are moral emotions [69,70,71]. Obviously, morality and emotion are intrinsically linked. Negative moral emotions triggered by a behavior or phenomenon violating moral cognitions stimulate moral identity; that is, negative message framing affects individual moral identity.

As a self-regulatory mechanism, moral identity motivates specific moral behaviors [40,41]. After receiving negative messages, individuals clearly recognize that littering is immoral and further adjust their behaviors to maintain self-consistency [72,73,74]. In this paper, we argue that moral identity motivated by negative framing enhances the willingness to separate. On the one hand, negative framing stimulates passive moral emotions and internalized moral identity by contradicting moral perceptions [75], which leads to a strong desire for individuals to maintain the inner harmony of their sense of self-worth [76,77]. On the other hand, not separating waste violates the moral traits that people want to display to the public or is inconsistent with the moral image they expect to build, which makes symbolization take effect. Therefore, people reduce behaviors that are inconsistent with the “external self” and are willing to separate waste. Hence, we propose the following hypotheses:

**Hypothesis** **3** **(H3).** *Negative message framing improves waste separation willingness by positively affecting internalization*.

**Hypothesis** **4** **(H4).** *Negative message framing improves waste separation willingness by positively affecting symbolization.*

### 2.3. Different Mediating Effects of Internalization and Symbolization

Although the theme or purpose of different framings is the same, the content and emotions expressed vary [78]. The primary motivation for symbolization is self-impression management, while internalization stems from maintaining self-consistency [34,79]. Therefore, message framings have different influences on the two dimensions of moral identity, causing different publicity effects [80].

Positive messages speak highly of the benefits and significance of waste separation, making people aware that separating is a moral action. It also suggests that separation will lead to recognition and praise from others, which enables people to gain a sense of extrinsic pride. In addition, pride is a self-conscious emotion that is characterized by focusing attention on self-representation [81,82], suggesting that individuals who are affected by positive message framing will have their moral identity symbolization regarding waste separation stimulated. Furthermore, when a behavior can be recognized by others, the predictive effect of symbolization is stronger than that of internalization [51,83,84]. Positive message framing divides waste separation into behaviors with public dimensions. External evaluations and recognitions strengthen individuals’ sense of self-identity and promote the role of symbolization [85], making the relationship between positive framing and symbolization stronger than that of internalization.

Wiltermuth et al. [86] found that symbolization is related to the praise of positive behaviors, while internalization is related to the condemnation of negative behaviors. Therefore, negative framing has a stronger relationship with internalization. At the same time, it causes people to empathize, which activates internalization [87,88]. Lee et al. [89] discovered that internalization influences charitable giving through empathy, while symbolization plays a less significant role in it. Through four experiments, Jiao et al. [87] concluded that loneliness and empathy affect only the internal dimension of moral identity and have no effect on the symbolic dimension. Empathy is more closely related to internalization than to symbolization. Moreover, the harmfulness presented by negative framing violates moral standards and causes guilt [90,91]. According to self-completion theory, although guilt is a negative emotion, it can motivate individuals to maintain their moral selves through prosocial behaviors [92,93], which is the same as the motivation of internalization. Zhang et al. [94] found that only the interaction of moral identity internalization and upward moral comparison has a significant impact on guilt, and people with a high degree of internalization are more likely to experience guilt. In summary, negative message framing is more strongly related to internalization than to symbolization.

When both dimensions of moral identity are activated, residents’ willingness to separate can be improved. However, due to the different strengths of the relationship between framings and the two dimensions, the mediating roles of internalization and symbolization appear to be differentiated. Based on the above related arguments, this paper proposes the following hypotheses:

**Hypothesis** **5** **(H5).** *The mediating role of symbolization in the relationship between positive message framing and waste separation willingness is stronger than internalization.*

**Hypothesis** **6** **(H6).** *The mediating role of internalization in the relationship between negative message framing and waste separation willingness is stronger than symbolization.*

## 3. Method

### 3.1. Participants

The purpose of this experiment was to explore the effects and differences in message framing on the willingness to separate waste mediated by two dimensions of moral identity. Based on the experimental design, we used the pwr package in R language to analyze the required sample size with a preset moderate effect size of f^2^ = 0.15 [95], statistical test power 1 − β = 0.8, and significance level α = 0.05, concluding that at least 77 subjects were needed in each group. To reduce between-group differences and social expectation bias, we increased the number of subjects. A total of 710 volunteers was recruited online through WeChat, Sina Weibo, etc., and were randomly assigned to the group that did not watch a message framing video (control group), the group that watched the positive message framing video, or the group that watched the negative message framing video (experimental group). After removing invalid data, 604 valid samples remained (290 males and 314 females), including 200 in the nonviewing group, 202 in the positive frame group, and 202 in the negative frame group. According to Table 1, the genders, ages, and monthly incomes of the subjects in three groups were roughly the same; in terms of education level, all groups had a higher ratio of junior college and university undergraduates, followed by high school or technical secondary school graduates, and then postgraduates and above, with junior high school graduates and below accounting for the lowest proportion, which was consistent with the educational structure of Chinese residents.

### 3.2. Design and Procedure

This study adopted a single-factor between-group design. The independent variable was message framing (0: no video; 1: positive message framing; 2: negative message framing), the mediating variables were internalization and symbolization of moral identity, and the dependent variable was waste separation willingness. Among them, message framing was an operating variable, while moral identity and waste separation willingness were measured variables. First, to hide the purpose of the experiment from the subjects and avoid bias when filling out the questionnaire, before the experiment, the subjects were informed that the purpose of the experiment was to explore the influence of aroused emotions on individuals’ dilemma decision making under the specific situation about waste separation. Second, the two experimental groups were asked to watch either a positively or negatively framed video, and the control group did not undergo the experimental process. Third, all three groups of subjects completed the questionnaire to test their moral identity level and separation willingness. Finally, after completing the questionnaire, the subjects were informed that the real purpose of this study was to investigate their waste separation willingness.

### 3.3. Experimental Manipulation and Variable Measurement

#### 3.3.1. Experimental Manipulation of Message Framing

Information such as pictures, text, and music can induce positive and negative emotions or enhance cognition [96,97,98]. The positively and negatively framed videos both consisted of text, pictures, and music. The specific materials were as follows:

The text part of the positively framed video consisted entirely of affirmative messages. For example, the information that reflected environmental benefits was “Separation can improve the resource value and utilization rate of waste. For example, plants and fabrics can be composted to produce organic fertilizers”. The information that showed personal benefits included “Waste separation conforms to professional code of ethics”. Related slogans included “Separating waste takes you and me one step closer to morality”. The pictures were divided into two categories: the reuse of waste, and residents’ separation behaviors. The background music was the lighter pure song, Home Again, by Jacques Davidovici.

The text part of the negatively framed video included negative warning messages. For example, the information showing environmental hazards was “some substances in household waste do not degrade easily and seriously eroded the land”. The information that reflected threats to human health was “Waste from house renovation can cause headache, allergy, coma and even cancer”. The warning messages included “No one can save our homes but ourselves”. Pictures included four parts: untreated waste, polluted environment, injured animals, and sick people. The background music was a powerful and deep absolute song, Lost but Won, by Hans Zimmer.

In the production of the two videos, we tried to keep all parts consistent to minimize errors. The positive video contained 248 words and 29 pictures and was 129 s long, and the negative video contained 315 words and 29 pictures and was 125 s long.

#### 3.3.2. Moral Identity

We referred to the scale compiled by Aquino and Reed [34]. Considering that the focus of this study was residents’ waste separation willingness, we revised it and adopted eight items, rated on a 5-point Likert scale, ranging from “very inconsistent” = 1 to “very consistent” = 5. The four items for symbolization were as follows: “Waste separation is consistent with my professional requirements and social identity”, “I hope that the fact that I actively participate in waste separating will be communicated to others by my colleagues in the organization”, “I want to show others that I am someone who can separate waste through my daily behaviors”, and “I would like to share with others the waste sorting activities and related knowledge”. The four items for internalization are as follows: “Sorting waste as required makes me feel that I am a moral person”, “I can realize my self-worth by sorting waste, and it is an important part of my personal character”, “I’d be very ashamed if I threw out rubbish without sorting it”, and “I am willing to sort waste, which improves my self-identity”. The Cronbach’s α reliability coefficients for the symbolization and internalization scales were 0.810 and 0.797, respectively.

#### 3.3.3. Waste Separation Willingness

When individuals describe or evaluate themselves, they tend to beautify themselves or overstate the fact, which results in deviations between the results of measurements and facts. In addition, willingness refers to the extent to which an individual has a positive inclination towards a certain thing, expressing an individual’s subjective attitude and intention. Therefore, to reduce experimental errors, we designed situational decision questions related to waste separation based on the scale compiled by Ajzen [99] to measure the participants’ separation willingness. The revised scale contains five items, each corresponding to five specific solutions. The gaps between the options are small, and there is no right or wrong. The items are as follows: “The bus you want to take comes every 15 min. The bus stop is very crowded and trash cans are 50 m away from it. You have some used napkins and packaging, but the bus is coming right away. What would you do?”; “Your community has started to implement the fixed-point waste disposal system, but the drop-off time conflicts with your work schedule. What would you do?”; “You buy a cup of bubble tea that does not suit your taste, then you want to throw away the rest. What would you do?”; “You order seafood takeaway. What will you do after eating?”; and “Your community is going to hold a publicity activity on the theme of ‘garbage classification in the community, green environmental protection in people’s heart’. What will you do?”. The Cronbach’s α reliability coefficients of this scale was 0.832.

#### 3.3.4. Control Variables

Based on the results of the meta-analysis by Kish-Gephart et al. [100], gender, age, education level, and monthly income were used as control variables in this study.

## 4. Results and Analysis

### 4.1. Correlation Analysis

Using the Amos 24.0 test scales, we found that the overall model fit well (χ^2^/df = 2.352, RMSEA = 0.05, CFI, IFI, TLI, NFI, and RFI were all greater than 0.9), and then we summarized the means, standard deviations, and correlation coefficients of the variables by SPSS24.0 (Table 2). The results showed that not viewing the video was significantly negative correlated with internalization and symbolization (mediating variables) (*p* < 0.01) and with waste separation willingness (dependent variable) (*p* < 0.01). The correlation coefficient of the positive message framing video with symbolization was 0.270 (*p* < 0.01), and that with separation willingness was 0.118 (*p* < 0.05); however, it was not significant for internalization. The correlation coefficients of negative message framing with internalization and separation willingness were both below 0.3 (*p* < 0.01), but with symbolization, they were insignificant. The correlation coefficients of the two mediating variables with separation willingness were both higher than 0.7 (*p* < 0.01). In summary, the correlation analysis results provide preliminary support for the hypothetical model.

### 4.2. The Effect of Message Framing on Waste Separation Willingness

Table 3 shows the single factor ANOVA test of separation willingness under the various frames. The means of waste separation willingness for the three groups were 3.394, 3.803, and 3.915, reaching significance at *p* < 0.001, indicating that message framing could improve separation willingness. In addition, the results of the LSD post hoc test depicted that the mean separation willingness of the group who did not view a video was lower than that of the other groups, and there was no significant difference in the separation willingness of the two experimental groups.

### 4.3. The Effect of Message Framing on Moral Identity

Table 4 presents the effects of message framing on moral identity. For internalization, the averages of the not viewing, positive frame, and negative frame groups were 3.786, 3.917, and 4.207, respectively, and passed the significance test at the 0.05 level. From the LSD post hoc test, the order of means was: negative frame > positive frame > not viewing (*p* < 0.05), showing that the internalization of the negative frame group was higher than that of the positive frame group; that is, negative message framing was more capable of improving internalization. For symbolization, the means of the not viewing, positive frame, and negative frame groups were 3.713, 4.129, and 3.878 (*p* < 0.05), respectively. The LSD post hoc test showed the order of the three means to be: positive frame > negative frame > not viewing (*p* < 0.05), suggesting that the symbolization of the positive frame group was higher than that of the negative frame group; that is, positive message framing was more able to improve the symbolization of residents.

### 4.4. The Mediating Effect of Moral Identity

Controlling for gender, age, education level, and monthly income, we adopted Model 4 in the SPSS macro developed by Hayes [101] to conduct a bootstrap mediation test with a sample size of 5000 [102], with message framing as the independent variable, moral identity as the mediating variable, and waste separation willingness as the dependent variable. Table 5 shows that the predictive effect of positive message framing on separation willingness was significant (*B* = 0.693, *t* = 7.482, *p* < 0.001), and after adding mediating variables, the direct predictive effect remained significant (*B* = 0.256, *t* = 4.948, *p* < 0.001). Positive framing had a significant positive impact on internalization (*B* = 0.221, *t* = 2.313, *p* < 0.05), and internalization had a significant positive effect on separation willingness (*B* = 0.392, *t* = 12.684, *p* < 0.001). Moreover, positive framing had a significant positive effect on symbolization (*B* = 0.713, *t* = 7.446, *p* < 0.001), and symbolization had a significant positive impact on separation willingness (*B* = 0.492, *t* = 15.927, *p* < 0.001). In addition, as seen in Table 6, the bootstrap 95% confidence intervals for the direct effect of positive frame on separation willingness and the mediating effect (Mediator 1: Internalization; Mediator 2: Symbolization) were [0.089, 0.216], [0.004, 0.104], and [0.147, 0.269], and the lower and upper confidence limits were both greater than zero. This indicates that positive message framing could not only directly predict separation willingness but also positively influence it through moral identity. H1 and H2 are supported by the data.

As shown in Table 5, the predictive effect of negative message framing on separation willingness was significant (*B* = 0.884, *t* = 9.548, *p* < 0.001), and after adding mediating variables, the direct predictive effect of negative framing on separation willingness remained significant (*B* = 0.467, *t* = 9.084, *p* < 0.001). Negative framing had a significant positive effect on internalization (*B* = 0.706, *t* = 7.395, *p* < 0.001), and internalization had a significant positive impact on separation willingness (*B* = 0.392, *t* = 12.684, *p* < 0.001). At the same time, negative framing had a significant predictive effect on symbolization (*B* = 0.286, *t* = 2.985, *p* < 0.01), and symbolization had a significant positive impact on separation willingness (*B* = 0.492, *t* = 15.927, *p* < 0.001). Table 7 shows that the bootstrap 95% confidence intervals for the direct effect of negative framing on separation willingness and the mediating effect (Mediator 1: Internalization; Mediator 2: Symbolization) were [0.222, 0.330], [0.117, 0.215], and [0.026, 0.141], respectively, and the lower and upper confidence limits were both higher than zero. Thus, negative message framing could not only directly predict separation willingness but also positively affected it through moral identity. H3 and H4 are supported by the data.

Table 6 shows that the total effect of positive message framing was 0.409. The mediating effect of internalization and symbolization was 0.051 and 0.207 and accounted for 12.51%, and 50.46% of the total effects, respectively. It can be seen that the mediating effect of symbolization accounted for a higher proportion than did internalization; that is, symbolization played a stronger mediating role in the relationship between positive message framing and waste separation willingness.

Moreover, Table 7 presents that the total effect of negative message framing was 0.522. The mediating effects of internalization and symbolization were 0.163 and 0.083, respectively, and account for 31.30% and 15.90%, respectively, of the total effects. The mediating effect of internalization had a higher effect ratio than symbolization, indicating that internalization had a stronger mediating role in the relationship between negative message framing and waste separation willingness.

In conclusion, H5 and H6 are supported by the data.

## 5. Discussion

1. Different effects of message framing on waste separation willingness. In the face of an increasingly severe environment, waste separation has become a topic of concern for all sectors of society, and a message strategy is an important means to popularize separation knowledge and promote residents’ willingness to separate [31,103,104,105]. Hu and Ning [106] found that the effect of publicity slogans classified as “for me” and “for us” according to beneficiaries on separation willingness vary with group size. In this study, we divided message strategies into positive and negative frames from the perspective of information expression and concluded that both frames increased separation willingness. Moreover, in the case of the same video structure, compared with the positive frame, negative message framing increased separation willingness to a greater extent, which is consistent with many findings on the differential effects of positive and negative information on behavioral willingness [22,66,107]. It is also in line with the goal framing effect proposed by Levin et al. [44], where using a negative frame (emphasizing that not doing something may result in a negative outcome) is more persuasive than using a positive frame.
2. The mediating role of moral identity between message framing and waste separation willingness. Based on social cognitive theory, this study introduced moral identity as a mediating variable and demonstrated that message framing not only directly influenced separation willingness but also enhanced it through moral identity. In the experimental manipulation of moral identity, its induction methods were divided into self-recall and external stimulation. To evoke moral identity, some studies have asked subjects to recall their own relevant experiences around certain moral traits or imagine how a person with these traits thinks and acts [25,108,109,110,111,112,113]. Other studies have applied extrinsic stimuli to objects through scenario setting [114], picture viewing [87], course learning [28], and so on. We adopted the second method, namely video viewing. The results of the moral identity level test showed that after the subjects watched the message framing videos, the average moral identity level in both the positive and negative frame groups was significantly higher than that in the control group, indicating that moral identity was stimulated. In addition, moral identity affects individuals’ judgments of moral responsibility and promotes their pursuit of self-consistency and moral integrity, forcing them to act in a moral way [24,115,116]. The results of the mediating effect test indicate that stimulated moral identity has a significant effect on separation willingness.

To date, to uncover the “black box”, there have been many studies on the mediating mechanism by which a message strategy affects behavior. Based on the theory of planned behavior, Liu et al. [117] explored the mediating role of attitudes, subjective norms, and perceived behavioral control in the relationship between public education and residents’ waste separation willingness and confirmed conscientious personality to moderate the relationship between public education and perceived behavioral control. Li et al. [118] found that two different environmental emotions, namely, guilt for not separating and pride in separating, mediate the relationship between public education and separation willingness. Based on existing studies, this study proves the mediating role of moral identity and enriches the research on the mechanism of message strategy.

3. Differences in the mediating role of internalization and symbolization. We also found that positive message framing had a greater impact on symbolization, and negative message framing had a greater effect on internalization. When external stimuli increase the accessibility of moral identity in the working self-concept, people are more motivated to act morally. Conversely, when the current accessibility of moral identity decreases, the motivation to act in a moral way decreases [25,109]. Several previous studies have also verified this relationship. For example, Mazar et al. [119] found that when moral identity is activated, subjects’ cheating behavior decreases or is eliminated. By analyzing the data from p2p lending websites, Herzenstein et al. [120] discovered that through the self-regulatory mechanism of moral identity, borrowers who were shown moral identity statements were more likely to repay on time. Undoubtedly, watching message framing videos increases the accessibility of moral identity in the working self-concept, and what matters is the matching degree between message framings and moral identity dimensions.

The symbolic and internal dimensions of moral identity correspond to the public self and inner self in personal identity, respectively [34,41,121,122]. Most studies have shown that symbolization is a stronger predictor of public behavior [123], whereas regardless of whether the expected behavior has public or private attributes, internalization maintains individual behavior based on self-consistency [84,124,125]. Positive message framing places waste separation into the public dimension. It praises waste separation while implying that it can present self-image to the public well, which has a stronger effect on moral identity symbolization. On the other hand, messages shown by negative message framing violate the consistency between moral identity and the self. Therefore, it improves willingness through the internal dimension to a greater extent [126]. However, Gotowiec and van Mastrigt [113] divided prosocial behavior into four domains (donation of time, effort, resources; civic engagement; prosociality in groups; and emotional responding) and found that after moral self-schemas are stimulated, symbolization has significant effects on both public and private behaviors, while the effect of internalization is not significant. We argue that this difference lies in the way moral self-schemas are activated. Gotowiec and van Mastrigt [113] used the self-recall method and asked subjects to complete in scales based on their previous behaviors, which were pre-existing. In contrast, different framings were used to stimulate moral identity based on different dimensions of motivation. Message framing makes individuals moralize waste separation and affects their judgments of whether waste separation belongs to the public or personal domain.

The level of moral identity varies between people, and the two dimensions of individual moral identity are also different. When internalization is low, high symbolization can motivate individuals to engage in identifiable prosocial behaviors that provide opportunities to present moral qualities to others [34,44]. In contrast, regardless of whether symbolization is high or low and whether a certain prosocial behavior is identifiable, individuals with high internalization are inclined to engage in prosocial behaviors [84]. Clearly, the two dimensions of moral identity motivate prosocial behaviors under different conditions, and internalization is more stable and more easily plays a role [34], which explains why the effect of negative framing on separation willingness is higher than that of positive framing. It is suggested that different framing messages are appropriate for different groups. For people with high internalization, negative framing is more likely to stimulate their desire to maintain behavior consistent with the self, while for groups with high symbolization, positive framing can better satisfy their needs to construct a self-image.

4. Limitations and prospects. Many factors, such as childhood environment, social experiences, and educational background, contribute to the formation of the unique social identities of individuals. Moral identity is a social identity that constitutes an individual’s social self-schema. Although it tends to be relatively stable over time, it can be activated or inhibited by situations [34,127]. Moral identity, which has long been thought to be the center of the self-concept, exists in long-term memory, while temporarily accessible moral identity is more like the information stored in working memory [110] and is more likely to affect immediate behaviors. It is certain that viewing the message framing materials stimulated the subjects’ moral identities, resulting in significantly higher levels of moral identity and separation willingness in the experimental groups than in the control group. However, it is not known how long the effect of temporary stimulation will last, depending on both the original level of moral identity and stimulation frequency. For individuals with a low level of moral identity, separation willingness was increased in the short term after viewing message framing, but can the “real” moral level be improved if individuals are educated for a long time? This should be explored in a long-term intertemporal experiment, which this study was unable to do. The subjects were recruited and tested online, and to ensure the randomness of the three groups, the number of subjects was greatly increased over the basic requirement, which made it difficult to operate and control the experiment over time. In the future, we will consider how to design long-term face-to-face experiments to explore this issue.

In addition, taking action is the ultimate goal of message framing. There is a clear gap between willingness and behavior in various moral dilemmas [128,129,130]. Through message framing, separation willingness has been improved, but whether it can be transformed into separation behavior depends on many factors, such as policies and infrastructure [46,131,132,133]. To make the message strategy work more effectively, it is very important to include behavioral research in future studies.

## 6. Conclusions

Based on moral identity theory from the perspective of social cognition, using moral identity as a mediating variable, we explored the mechanism of message framing on waste separation willingness and expanded research in this field. In recent years, natural disasters and viral spreading have threatened human health, which warn us of the importance of environmental protection. Therefore, it is particularly important to explore how residents’ separation willingness can be increased. The results demonstrate that message framing is an important means of improving separation willingness, and moral identity explains why message framing has different effects on separation willingness, providing suggestions for carrying out a specific message strategy. Positive message framing plays a greater role in groups with high symbolization, such as community residents and corporate staff, while negative message framing is more effective among groups with high internalization, such as university teachers and students. In other words, message framing can be most effective when aimed at specific groups. Moreover, the improvement in moral literacy is the key to increasing the willingness of green behaviors, which requires not only extensive information publicity by governments but also organized ideological education within groups to improve the level of personal moral identity and develop a good habit of waste separation.

## Figures and Tables

**Table 1:** Descriptive statistics of demographic variables.

Variables	Items	No Viewing		Positive Frame		Negative Frame	
		Frequency	Percentage	Frequency	Percentage	Frequency	Percentage
Gender	Male	94	47.0	97	48.0	99	49.0
	Female	106	53.0	105	52.0	103	51.0
Age	Under 25	52	26.0	61	30.2	59	29.2
	26–35	53	26.5	52	25.7	54	26.7
	36–45	53	26.5	48	23.8	47	23.3
	Above 45	42	21.0	41	20.3	42	20.8
Education level	Junior high school or below	4	2.0	3	1.5	5	2.5
	High school or technical secondary school	21	10.5	17	8.4	19	9.4
	Junior college	42	21.0	40	19.8	46	22.8
	University	106	53.0	114	56.4	102	50.5
	Master’s degree or above	27	13.5	28	13.9	30	14.9
Monthly income (RMB)	Below 3000	35	17.5	46	22.8	37	18.3
	3001–5000	61	30.5	60	29.7	68	33.7
	5001–8000	70	35.0	54	26.7	61	30.2
	8001–12,000	26	13.0	33	16.3	29	14.4
	Above 12,000	8	4.0	9	4.5	7	3.5

**Table 2:** Mean, standard deviation, and correlation.

Variables	1	2	3	4	5	6	7	8	9	10
1 Gender	1									
2 Age	0.008	1								
3 Education level	0.027	−0.339 **	1							
4 Monthly income	−0.054	0.434 **	0.069	1						
5 Not viewing	0.014	0.032	−0.020	0.022	1					
6 Positive frame	0.000	−0.021	0.037	−0.014	−0.499 **	1				
7 Negative frame	−0.014	−0.011	−0.017	−0.008	−0.499 **	−0.502 **	1			
8 Internalization	0.076	0.004	0.004	0.010	−0.217 **	−0.063	0.280 **	1		
9 Symbolization	0.008	0.033	0.030	−0.016	−0.234 **	0.270 **	−0.036	0.687 **	1	
10 Separation willingness	0.026	0.005	−0.001	−0.046	−0.371 **	0.118 *	0.252 **	0.783 **	0.786 **	1
Mean	1.520	2.374	3.680	2.522	0.331	0.334	0.334	3.971	3.907	3.705
Standard deviation	0.500	1.104	0.899	1.083	0.471	0.472	0.472	0.599	0.584	0.590

N = 604, ** *p* < 0.01, * *p* < 0.05.

**Table 3:** ANOVA test of separation willingness under different message framing.

Group	Separation Willingness	
	Mean	Standard Deviation
Not viewing	3.786	0.674
Positive frame	3.917	0.589
Negative frame	4.207	0.432
F	28.348 ***	
LSD	0 < 1 < 2	

*** *p* < 0.001.

**Table 4:** ANOVA of moral identity under different message framing.

Group	Internalization		Symbolization	
	Mean	Standard Deviation	Mean	Standard Deviation
Not viewing	3.786	0.674	3.713	0.689
Positive frame	3.917	0.589	4.129	0.478
Negative frame	4.207	0.432	3.878	0.486
F	28.348 ***		28.261 ***	
LSD	0 < 1 < 2		0 < 2 < 1	

*** *p* < 0.001.

**Table 5:** Mediation model test.

	Separation Willingness		Separation Willingness		Internalization		Symbolization	
	*B*	*t*	*B*	*t*	*B*	*t*	*B*	*t*
Positive frame	0.693	7.482 ***	0.256	4.948 ***	0.221	2.313 *	0.713	7.446 ***
Negative frame	0.884	9.548 ***	0.467	9.084 ***	0.706	7.395 ***	0.286	2.985 **
Gender	0.028	0.744	−0.008	−0.333	0.082	2.086 *	0.006	0.151
Age	0.046	0.997	0.003	0.242	0.003	0.072	0.079	1.654
Education level	0.013	0.306	−0.009	−0.599	0.004	0.088	0.050	1.155
Monthly income	−0.057	−1.318	−0.022	−1.780	0.017	0.378	−0.047	−1.047
Internalization			0.392	12.684 ***				
Symbolization			0.492	15.927 ***				
R	0.384		0.875		0.305		0.301	
R^2^	0.148		0.765		0.093		0.091	
F	17.220 ***		242.174 ***		10.213 ***		9.929 ***	

*** *p* < 0.001, ** *p* < 0.01, * *p* < 0.05.

**Table 6:** The total effect, direct effect, and mediating effect of positive message framing.

		Effect	BootSE	BootLLCL	BootULCI	Relative Effect Value
Total effect		0.409	0.058	0.295	0.523	
Direct effect		0.151	0.032	0.089	0.216	36.93%
Mediating effect	IN ^1^	0.051	0.025	0.004	0.104	12.51%
	SY ^2^	0.207	0.031	0.147	0.269	50.46%

^1^ Internalization, ^2^ Symbolization.

**Table 7:** The total effect, direct effect, and mediating effect of negative message framing.

		Effect	BootSE	BootLLCL	BootULCI	Relative Effect Value
Total effect		0.522	0.057	0.414	0.635	
Direct effect		0.276	0.028	0.222	0.330	52.80%
Mediating effect	IN ^1^	0.163	0.025	0.117	0.215	31.30%
	SY ^2^	0.083	0.030	0.026	0.141	15.90%

^1^ Internalization, ^2^ Symbolization.

## Data Availability

Not applicable.

## References

[1] Grazhdani D. (2016). Assessing the variables affecting on the rate of solid waste generation and recycling: An empirical analysis in Prespa Park. Waste Manag..

[2] Knutsson S.G., Asplund T., Höst G., Schönborn K.J. (2021). Public perceptions of waste management in Sri Lanka: A focus group study. Sustainability.

[3] Gu B., Zhu W., Wang H., Zhang R., Liu M., Chen Y., Wu Y., Yang X., He S., Cheng R. (2014). Household hazardous waste quantification, characterization and management in China’s cities: A case study of Suzhou. Waste Manag..

[4] Tobler C., Visschers V.H.M., Siegrist M. (2012). Addressing climate change: Determinants of consumers′ willingness to act and to support policy measures. J. Environ. Psychol..

[5] Colon M., Fawcett B. (2006). Community-based household waste management: Lessons learnt from EXNORA′s ‘zero waste management’ scheme in two South Indian cities. Habitat Int..

[6] Zurbrügg C., Drescher S., Patel A., Sharatchandra H.C. (2004). Decentralised composting of urban waste—An overview of community and private initiatives in Indian cities. Waste Manag..

[7] Liu Q., Xu Q., Shen X., Chen B., Esfahani S.S. (2022). The mechanism of household waste sorting behaviour—A study of Jiaxing, China. Int. J. Environ. Res. Public Health.

[8] Schultz P.W., Oskamp S., Mainieri T. (1995). Who recycles and when? A review of personal and situational factors. J. Environ. Psychol..

[9] Wang H., Liu X., Wang N., Zhang K., Wang F., Zhang S., Wang R., Zheng P., Matsushita M. (2020). Key factors influencing public awareness of household solid waste recycling in urban areas of China: A case study. Resour. Conserv. Recycl..

[10] Liao C., Li H. (2019). Environmental education, knowledge, and high school students’ intention toward separation of solid waste on campus. Int. J. Environ. Res. Public Health.

[11] Fujiki H. (2016). The sorting process of construction wastes in Japan. Ann. Soc. Ind. Stud..

[12] Kang L., Zhu K. (2019). Enlightenment of waste classification management experience in Japan and germany to waste classification work in China. J. EMCC.

[13] Massarutto A., Marangon F., Troiano S., Favot M. (2018). Moral duty, warm glow or self-interest? A choice experiment study on motivations for domestic garbage sorting in Italy. J. Clean. Prod..

[14] Zhu H., Ao Y., Xu H., Zhou Z., Wang Y., Yang L. (2021). Determinants of farmers’ intention of straw recycling: A comparison analysis based on different pro-environmental publicity modes. Int. J. Environ. Res. Public Health.

[15] Zeng S., Zhou Y. (2021). Foreign direct investment′s impact on China′s economic growth, technological innovation and pollution. Int. J. Environ. Res. Public Health.

[16] Chen D., Wang Y., Wen Y., Du H., Tan X., Shi L., Ma Z. (2021). Does environmental policy help green industry? Evidence from China’s promotion of municipal solid waste sorting. Int. J. Environ. Res. Public Health.

[17] Chen J., Lin W., Li Y. (2020). An empirical study on the intention and behavior of garbage classification: A case study of Guangzhou. Urban Insight.

[18] Treviño L.K., Weaver G.R., Reynolds S.J. (2006). Behavioral ethics in organizations: A review. J. Manag..

[19] Razali F., Daud D., Weng-Wai C., Jiram W.R.A. (2020). Waste separation at source behaviour among Malaysian households: The Theory of Planned Behaviour with moral norm. J. Clean. Prod..

[20] Kim Y.H., Son S.Y., Kang S.W. (2021). Effects of anger and moral identity on the relationship between supervisors’ incivility and deviant behavior: A study of public service officers in Republic of Korea. Int. J. Environ. Res. Public Health.

[21] Zeng S., Li G., Wu S., Dong Z. (2022). The impact of green technology innovation on carbon emissions in the context of carbon neutrality in China: Evidence from spatial spillover and nonlinear effect analysis. Int. J. Environ. Res. Public Health.

[22] Amatulli C., De Angelis M., Peluso A.M., Soscia I., Guido G. (2017). The effect of negative message framing on green consumption: An investigation of the role of shame. J. Bus. Ethics.

[23] Skitka L.J., Bauman C.W., Sargis E.G. (2005). Moral conviction: Another contributor to attitude strength or something more?. J. Pers. Soc. Psychol..

[24] Blasi A. (1983). Moral cognition and moral action: A theoretical perspective. Dev. Rev..

[25] Aquino K., Freeman D., Reed A., Lim V.K.G., Felps W. (2009). Testing a social-cognitive model of moral behavior: The interactive influence of situations and moral identity centrality. J. Pers. Soc. Psychol..

[26] Rozin P., Singh L. (1999). The moralization of cigarette smoking in the United States. J. Consum. Psychol..

[27] Smith C.P., Ryan E.R., Diggins D.R. (1972). Moral decision making: Cheating on examinations. J. Pers..

[28] Feinberg M., Kovacheff C., Teper R., Inbar Y. (2019). Understanding the process of moralization: How eating meat becomes a moral issue. J. Pers. Soc. Psychol..

[29] Pakpour A.H., Zeidi I.M., Emamjomeh M.M., Asefzadeh S., Pearson H. (2014). Household waste behaviours among a community sample in Iran: An application of the theory of planned behaviour. Waste Manag..

[30] Chan L., Bishop B. (2013). A moral basis for recycling: Extending the theory of planned behaviour. J. Environ. Psychol..

[31] Qi S., Zhang C. (2019). Research on current situation of urban domestic waste classification and its countermeasures—Based on the survey of C city in Shanxi Province. Sci. Tech. Innov. Prod..

[32] Ferguson E., Gallagher L. (2007). Message framing with respect to decisions about vaccination: The roles of frame valence, frame method and perceived risk. Br. J. Psychol..

[33] Suk W.A., Ahanchian H., Asante K.A., Carpenter D.O., Diaz-Barriga F., Ha E.H., Huo X., King M., Ruchirawat M., da Silva E.R. (2016). Environmental pollution: An under-recognized threat to children′s health, especially in low- and middle-income countries. Environ. Health Perspect..

[34] Aquino K., Reed A. (2002). The self-importance of moral identity. J. Pers. Soc. Psychol..

[35] Wu B., Li D., Wang C. (2016). The psychological mechanism of green consumption based on moral identity theory. Adv. Psychol. Sci..

[36] Wang Z., Guo D., Wang X., Zhang B., Wang B. (2018). How does information publicity influence residents’ behaviour intentions around e-waste recycling?. Resour. Conserv. Recycl..

[37] Cui T., Su X., Zhang Y. (2021). Study on compulsory classification management and behavior synergy of municipal solid waste. Sustainability.

[38] Shao R., Aquino K., Freeman D. (2008). Beyond moral reasoning: A review of moral identity research and its implications for business ethics. Bus. Ethics Q..

[39] Stets J.E., Carter M.J. (2011). The moral self. Soc. Psychol. Q..

[40] Blasi A., Kurtines W.M., Gewirtz J.L. (1985). Moral Identity: Its Role in Moral Functioning. Morality, Moral Behavior and Moral Development.

[41] Erikson E.H. (1964). Insight and Responsibility: Lectures on the Ethical Implications of Psychoanalytic Insight. Ethics.

[42] Barkan R., Ayal S., Ariely D. (2015). Ethical dissonance, justifications, and moral behavior. Curr. Opin. Psychol..

[43] Bamberg S., Möser G. (2007). Twenty years after hines, hungerford, and tomera: A new meta-analysis of psycho-social determinants of pro-environmental behaviour. J. Environ. Psychol..

[44] Winterich K.P., Aquino K., Mittal V., Swartz R. (2013). When moral identity symbolization motivates prosocial behavior: The role of recognition and moral identity internalization. J. Appl. Psychol..

[45] Wan Z. (2008). The Psychological Development and The Construct of Moral Identity.

[46] Chen S., Li R., Ma Y. (2015). Paradox between willingness and behavior: Classification mechanism of urban residents on household waste. China Popul. Resour. Environ..

[47] Boonrod K., Towprayoon S., Bonnet S., Tripetchkul S. (2015). Enhancing organic waste separation at the source behavior: A case study of the application of motivation mechanisms in communities in Thailand. Resour. Conserv. Recycl..

[48] Xia Y., Liu Y., Han C., Gao Y., Lan Y. (2022). How does environmentally specific servant leadership fuel employees′ low-carbon behavior? The role of environmental self-accountability and power distance orientation. Int. J. Environ. Res. Public Health.

[49] Hart D., Carlo G., Edwards C.P. (2005). The Development of Moral Identity. Nebraska Symposium on Motivation: Moral Development through the Lifespan: Theory, Research, and Application.

[50] McDonagh E.L. (1982). Social exchange and moral development: Dimensions of self, self-image, and identity. Hum. Relat..

[51] Barclay L.J., Whiteside D.B., Aquino K. (2014). To avenge or not to avenge? Exploring the interactive effects of moral identity and the negative reciprocity norm. J. Bus. Ethics.

[52] Schlenker B.R. (1980). Impression Management: The Self-Concept, Social Identity, and Interpersonal Relations.

[53] Hardy S.A., Carlo G. (2005). Identity as a source of moral motivation. Hum. Dev..

[54] Grant A.M. (2012). Giving time, time after time: Work design and sustained employee participation in corporate volunteering. Acad. Manag. Rev..

[55] Shen J., Zheng D., Zhang X., Qu M. (2020). Investigating rural domestic waste sorting intentions based on an integrative framework of planned behavior theory and normative activation models: Evidence from guanzhong basin, China. Int. J. Environ. Res. Public Health.

[56] Felson B.R. (1978). Aggression as impression management. Soc. Psychol..

[57] Youniss J., Yates M. (1997). Community Service and Social Responsibility in Youth.

[58] Rasmussen D.M., Habermas J., Lenhardt C., Nicholsen S.W. (1993). Moral consciousness and communicative action. Philos. Q..

[59] Fu X., Lu Z., Kou Y. (2015). Effects of a stranger′s presence and behavior on moral hypocrisy. Acta Psychol. Sin..

[60] Gerend M.A., Cullen M. (2008). Effects of message framing and temporal context on college student drinking behavior. J. Exp. Soc. Psychol..

[61] Wistar A., Hall M.G., Bercholz M., Taillie L.S. (2022). Designing environmental messages to discourage red meat consumption: An online experiment. Int. J. Environ. Res. Public Health.

[62] Chang C.T., Lee Y.K. (2009). Framing charity advertising: Influences of message framing, image valence, and temporal framing on a charitable appeal. J. Appl. Soc. Psychol..

[63] Triassi M., Alfano R., Illario M., Nardone A., Caporale O., Montuori P. (2015). Environmental pollution from illegal waste disposal and health effects: A review on the “triangle of death”. Int. J. Environ. Res. Public Health.

[64] Taylor S.E. (1991). Asymmetrical effects of positive and negative events: The mobilization-minimization hypothesis. Psychol. Bull..

[65] Davis J.J. (1995). The effects of message framing on response to environmental communications. J. Mass Commun. Q..

[66] Olsen M.C., Slotegraaf R.J., Chandukala S.R. (2014). Green claims and message frames: How green new products change brand attitude. J. Mark..

[67] Grob A. (1995). A structural model of environmental attitudes and behaviour. J. Environ. Psychol..

[68] Tapia-Fonllem C., Corral-Verdugo V., Fraijo-Sing B., Durón-Ramos M. (2013). Assessing sustainable behavior and its correlates: A measure of pro-ecological, frugal, altruistic and equitable actions. Sustainability.

[69] Haidt J., Davidson R.J., Scherer K.R., Goldsmith H.H. (2003). The moral emotions. Handbook of Affective Sciences.

[70] Weiner B. (2006). Social Motivation, Justice, and the Moral Emotions: An Attributional Approach.

[71] Lee H., An S., Lim G.Y., Sohn Y.W. (2021). Ethical leadership and followers’ emotional exhaustion: Exploring the roles of three types of emotional labor toward leaders in South Korea. Int. J. Environ. Res. Public Health.

[72] Haidt J. (2001). The emotional dog and its rational tail: A social intuitionist approach to moral judgment. Psychol. Rev..

[73] Stets J.E., Michael J.C., McClelland K., Fararo T.J. (2006). The moral identity: A principle level identity. Purpose, Meaning, and Action: Control Systems Theories in Sociology.

[74] Zhang L., Ran W., Jiang S., Wu H., Yuan Z. (2021). Understanding consumers′ behavior intention of recycling mobile phone through formal channels in China: The effect of privacy concern. Resour. Environ. Sustain..

[75] Wisneski D.C., Skitka L.J. (2017). Moralization through moral shock: Exploring emotional antecedents to moral conviction. Pers. Soc. Psychol. Bull..

[76] Damon W., Gregory A. (1997). The youth charter: Towards the formation of adolescent moral identity. J. Moral Educ..

[77] Basil D.Z., Ridgway N.M., Basil M.D. (2010). Guilt and giving: A process model of empathy and efficacy. Psychol. Mark..

[78] Tversky A., Kahneman D. (1981). The framing of decisions and the psychology of choice. Science.

[79] Kristofferson K., White K., Peloza J. (2014). The nature of slacktivism: How the social observability of an initial act of token support affects subsequent prosocial action. J. Consum. Res..

[80] Chang M.C., Wu C.C. (2015). The effect of message framing on pro-environmental behavior intentions. Br. Food J..

[81] Tracy J.L., Robins R.W. (2007). The psychological structure of pride: A tale of two facets. J. Pers. Soc. Psychol..

[82] Sanders S., Wisse B., Van Yperen N.W., Rus D. (2018). On ethically solvent leaders: The roles of pride and moral identity in predicting leader ethical behavior. J. Bus. Ethics.

[83] Hertz S.G., Krettenauer T. (2016). Does Moral Identity Effecttively Predict Moral Behavior?: A Meta-Analysis. Rev. Gen. Psychol..

[84] Winterich K.P., Mittal V., Aquino K. (2013). When does recognition increase charitable behavior? Toward a moral identity-based model. J. Mark..

[85] Lieberman M., Gauvin L., Bukowski W.M., White D.R. (2001). Interpersonal influence and disordered eating behaviors in adolescent girls. Eat. Behav..

[86] Wiltermuth S.S., Monin B., Chow R.M. (2010). The orthogonality of praise and condemnation in moral judgment. Soc. Psychol. Pers. Sci..

[87] Jiao J.J., Wang J. (2018). Can lonely people behave morally? The joint influence of loneliness and empathy on moral identity. J. Consum. Psychol..

[88] Yang H.T., Yen G.F. (2018). Consumer responses to corporate cause-related marketing: A serial multiple mediator model of self-construal, empathy and moral identity. Eur. J. Market..

[89] Lee S., Winterich K.P., Ross W.T. (2014). I′m moral, but i won′t help you: The distinct roles of empathy and justice in donations. J. Consum. Res..

[90] Tangney J.P., Stuewig J., Mashek D.J. (2007). Moral emotions and moral behavior. Annu. Rev. Psychol..

[91] Malti T., Ongley S.F., Killen M., Smetana J.G., Killen M., Smetana J.G. (2014). The development of moral emotions and moral reasoning. Handbook of Moral Development.

[92] Jordan J., Mullen E., Murnighan J.K. (2011). Striving for the moral self: The effects of recalling past moral actions on future moral behavior. Pers. Soc. Psychol. Bull..

[93] Rees J.H., Klug S., Bamberg S. (2015). Guilty conscience: Motivating pro-environmental behavior by inducing negative moral emotions. Clim. Chang..

[94] Zhang H., Chen S., Wang R., Jiang J., Xu Y., Zhao H. (2017). How upward moral comparison influences prosocial behavioral intention: Examining the mediating role of guilt and the moderating role of moral identity. Front. Psychol..

[95] Cohen J. (1988). Statistical Power Analysis for the Behavioral Sciences.

[96] Koelsch S. (2005). Investigating emotion with music: Neuroscientific approaches. Ann. N. Y. Acad. Sci..

[97] Dreisbach G. (2006). How positive affect modulates cognitive control: The costs and benefits of reduced maintenance capability. Brain Cogn..

[98] Yu Y., Jiang Y., Fang P., He Q., Zhang K. (2014). Measurement for music-induced emotions and its interacting factors. Stud. Psychol. Behav..

[99] Ajzen I. (1991). The theory of planned behavior. Organ. Behav. Hum. Decis. Process..

[100] Kish-Gephart J.J., Harrison D.A., Treviño L.K. (2010). Bad apples, bad cases, and bad barrels: Meta-analytic evidence about sources of unethical decisions at work. J. Appl. Psychol..

[101] Hayes A.F. (2012). PROCESS: A Versatile Computational Tool for Observed Variable Mediation, Moderation, and Conditional Process Modeling.

[102] Preacher K.J., Hayes A.F. (2004). SPSS and SAS procedures for estimating indirect effects in simple mediation models. Behav. Res. Methods Instrum. Comput..

[103] Ekere W., Mugisha J., Drake L. (2009). Factors influencing waste separation and utilization among households in the Lake Victoria crescent, Uganda. Waste Manag..

[104] Wan A., Rusli I.F., Biak D., Idris A. (2013). An application of the theory of planned behaviour to study the influencing factors of participation in source separation of food waste. Waste Manag..

[105] Chen F., Chen H., Long R., Long Q. (2017). Prediction of environmental cognition to undesired environmental behavior—the interaction effect of environmental context. Environ. Prog. Sustain. Energy.

[106] Hu C., Ning C. (2021). For “Me” or for “Us”? A study on the influences of different types of garbage classification propa-ganda on the willingness of garbage classification behavior. Forecasting.

[107] Abhyankar P., O’Connor D.B., Lawton R. (2008). The role of message framing in promoting MMR vaccination: Evidence of a loss-frame advantage. Psychol. Health Med..

[108] Reed A., Aquino K., Levy E. (2007). Moral identity and judgments of charitable behaviors. J. Mark..

[109] Aquino K., Reed A., Thau S., Freeman D. (2007). A grotesque and dark beauty: How moral identity and mechanisms of moral disengagement influence cognitive and emotional reactions to war. J. Exp. Soc. Psychol..

[110] Wu B. (2014). Moral Identity and Green Consumption: The Mediation Effect of Environmental Protection Self-Accountability.

[111] Krettenauer T., Murua L.A., Jia F. (2016). Age-related differences in moral identity across adulthood. Dev. Psychol..

[112] Jia F., Soucie K., Alisat S., Curtin D., Pratt M. (2017). Are environmental issues moral issues? Moral identity in relation to protecting the natural world. J. Environ. Psychol..

[113] Gotowiec S., van Mastrigt S. (2018). Having versus doing: The roles of moral identity internalization and symbolization for prosocial behaviors. J. Soc. Psychol..

[114] Reynolds S.J., Ceranic T.L. (2007). The effects of moral judgment and moral identity on moral behavior: An empirical examination of the moral individual. J. Appl. Psychol..

[115] Colby A., Damon W. (1992). Some do Care: Contemporary Lives of Moral Commitment.

[116] Schlenker B.R., Miller M.L., Johnson R.M., Narvaez D., Laplsey D.K. (2009). Moral identity, integrity, and personal responsibility. Personality, Identity, and Character: Explorations in Moral Psychology.

[117] Liu X., Wang Z., Li W., Li G., Zhang Y. (2019). Mechanisms of public education influencing waste classification willingness of urban residents. Resour. Conserv. Recycl..

[118] Li W., Wang Z., Liu X. (2021). Mechanisms of public education influencing waste sorting willingness of urban residents—The mediation of environment emotion and moderation of moral identity. J. Arid Land Resour. Environ..

[119] Mazar N., Amir O., Ariely D. (2008). The dishonesty of honest people: A theory of self-concept maintenance. J. Mark. Res..

[120] Herzenstein M., Sonenshein S., Dholakia U.M. (2011). Tell me a good story and i may lend you my money: The role of narratives in peer-to-peer lending decisions. J. Mark. Res..

[121] Fenigstein A., Scheier M.F., Buss A.H. (1975). Public and private self-consciousness: Assessment and theory. J. Consult. Clin. Psychol..

[122] Schlenker B.R., Weigold M.F. (1992). Interpersonal processes involving impression regulation and management. Annu. Rev. Psychol..

[123] Mayer D.M., Aquino K., Greenbaum R.L., Kuenzi M. (2012). Who displays ethical leadership, and why does it matter? An examination of antecedents and consequences of ethical leadership. Acad. Manag. J..

[124] Reed A., Aquino K.F. (2003). Moral identity and the expanding circle of moral regard toward out-groups. J. Pers. Soc. Psychol..

[125] Sage L., Kavussanu M., Duda J. (2006). Goal orientations and moral identity as predictors of prosocial and antisocial functioning in male association football players. J. Sports Sci..

[126] Hou J., Jin Y., Chen F. (2020). Should waste separation be mandatory? A study on public’s response to the policies in China. Int. J. Environ. Res. Public Health.

[127] Oyserman D. (2009). Identity-based motivation: Implications for action-readiness, procedural-readiness, and consumer behavior. J. Consum. Psychol..

[128] Epley N., Dunning D. (2000). Feeling “holier than thou”: Are self-serving assessments produced by errors in self- or social prediction?. J. Pers. Soc. Psychol..

[129] Teper R., Inzlicht M., Page-Gould E. (2011). Are we more moral than we think? Exploring the role of affect in moral behavior and moral forecasting. Psychol. Sci..

[130] Teper R., Zhong C.B., Inzlicht M. (2015). How emotions shape moral behavior: Some answers (and questions) for the field of moral psychology. Soc. Pers. Psychol. Compass.

[131] Batson C.D., Thompson E.R., Seuferling G., Whitney H., Strongman J.A. (1999). Moral hypocrisy: Appearing moral to oneself without being so. J. Pers. Soc. Psychol..

[132] Hao M., Zhang D., Morse S. (2020). Waste separation behaviour of college students under a mandatory policy in China: A case study of Zhengzhou City. Int. J. Environ. Res. Public Health.

[133] Yang S., Cheng P., Wang S., Li J. (2021). Towards sustainable cities: The spillover effects of waste-sorting policies on sustainable consumption. Int. J. Environ. Res. Public Health.

